# Teacher Competence in Mindfulness-Based Cognitive Therapy for Depression and Its Relation to Treatment Outcome

**DOI:** 10.1007/s12671-016-0672-z

**Published:** 2017-01-12

**Authors:** Marloes J. Huijbers, Rebecca S. Crane, Willem Kuyken, Lot Heijke, Ingrid van den Hout, A. Rogier T. Donders, Anne E.M. Speckens

**Affiliations:** 1 Department of Psychiatry, Radboud University Nijmegen Medical Centre, Reinier Postlaan 10, 6525 GC Nijmegen, The Netherlands; 2 Centre for Mindfulness Research and Practice, School of Psychology, Bangor University, Bangor, LL57 2AS UK; 3 Department of Psychiatry, Warneford Hospital, University of Oxford, Oxford, OX3 7JX UK; 4 Present Mind, Mindfulness Training and Education, 1053 RN Amsterdam, The Netherlands; 5 Outpatient Clinic for Mental Health, Dokter Bosman, Houttuinlaan 16A, 3447 GM Woerden, The Netherlands; 6 Department for Health Evidence, Radboud University Nijmegen Medical Centre, Geert Grooteplein 21, 6525 EZ Nijmegen, The Netherlands

**Keywords:** Mindfulness-based cognitive therapy, Recurrent depression, Intervention integrity, Therapist competence, Teacher competence

## Abstract

As mindfulness-based cognitive therapy (MBCT) becomes an increasingly mainstream approach for recurrent depression, there is a growing need for practitioners who are able to teach MBCT. The requirements for being competent as a mindfulness-based teacher include personal meditation practice and at least a year of additional professional training. This study is the first to investigate the relationship between MBCT teacher competence and several key dimensions of MBCT treatment outcomes. Patients with recurrent depression in remission (*N* = 241) participated in a multi-centre trial of MBCT, provided by 15 teachers. Teacher competence was assessed using the Mindfulness-Based Interventions: Teaching Assessment Criteria (MBI:TAC) based on two to four randomly selected video-recorded sessions of each of the 15 teachers, evaluated by 16 trained assessors. Results showed that teacher competence was not significantly associated with adherence (number of MBCT sessions attended), possible mechanisms of change (rumination, cognitive reactivity, mindfulness, and self-compassion), or key outcomes (depressive symptoms at post treatment and depressive relapse/recurrence during the 15-month follow-up). Thus, findings from the current study indicate no robust effects of teacher competence, as measured by the MBI:TAC, on possible mediators and outcome variables in MBCT for recurrent depression. Possible explanations are the standardized delivery of MBCT, the strong emphasis on self-reliance within the MBCT learning process, the importance of participant-related factors, the difficulties in assessing teacher competence, the absence of main treatment effects in terms of reducing depressive symptoms, and the relatively small selection of videotapes. Further work is required to systematically investigate these explanations.

## Introduction

Mindfulness-based cognitive therapy (MBCT) is an increasingly popular intervention in mental health care and beyond. It was developed by Segal et al. (2002b) as a relapse prevention method for patients with recurrent major depressive disorder (MDD). The evidence base of MBCT is growing, and a recent individual patient data meta-analysis suggests it is superior to usual care and at least as effective as other active treatments (Kuyken et al. 2016). It is recommended by guidelines on depression prevention such as those by the American Psychiatric Association (American Psychiatric Association 2006) and the National Institute for Health and Clinical Excellence (NICE) (National Institute for Clinical Excellence 2004) and the Dutch guidelines on evidence-based practice (Spijker et al. 2012). MBCT is based on the rationale that people become more vulnerable to developing depression when they have strong automatic patterns of (negative) thinking or behaving in response to a stressful event or a decrease in mood, referred to as cognitive reactivity (Scher et al. 2005). Cognitive reactivity often leads to a further lowering of mood, eventually turning into a depressive relapse/recurrence (Segal et al. 1999). In MBCT, participants learn to become aware of their automatic cognitive reactions to low mood or stress and to observe these reactions with kindness and curiosity (Segal et al. 2012). There is evidence that MBCT diminishes the ‘toxic’ relationship between cognitive reactivity and poor outcome—i.e. cognitive reactivity no longer predicted depression severity at 1-year follow-up in patients who participated in MBCT, in contrast with those receiving antidepressant medication (Kuyken et al. 2010). Other studies have suggested that rumination, which refers to the recurrent thinking about one’s own depressive symptoms and their possible causes and implications, may also be an important mediating factor (Hawley et al. 2014; Ramel et al. 2004; Van Aalderen et al. 2012b). In addition, evidence suggests that both mindfulness skills and self-compassion mediate the effect of MBCT on clinical outcome (Van der Velden et al. 2015).

Up to now, the teachers in the trials included in the meta-analysis on MBCT for recurrent MDD (Kuyken et al. 2016) were either the developers of MBCT or were trained by the developers of MBCT. However, the field is rapidly developing, with a growing need for qualified teachers. Concerns have been expressed about organizations or individuals responding to this need before engaging in or completing the required teacher training (Crane et al. 2010; Santorelli et al. 2011). In parallel, some consensus has emerged concerning minimum training standards and good practice guidelines (Kabat-Zinn et al. n.d.; UK Network of Mindfulness-Based Teacher Trainers 2010). Additionally, some measures have been developed to operationalize the adherence and competence of the teachers who deliver mindfulness-based interventions, such as the MBCT Adherence Scale (Segal et al. 2002a), the Mindfulness-Based Relapse Prevention (MBRP) Adherence and Competence Scale (Chawla et al. 2010), and the Mindfulness-Based Interventions: Teaching Assessment Criteria (MBI:TAC; Crane et al. 2013). However, research investigating the relationship between therapist competence in MBCT or mindfulness-based stress reduction (MBSR) and intervention outcomes is still lacking. In fact, “engaging the thorny question of clinician training” has been formulated as one of the important gaps in the current evidence base for mindfulness-based interventions (Dimidjian and Segal 2015, p. 605).

Therapist competence has been defined as “the extent to which a therapist has the knowledge and skill required to deliver a treatment to the standard needed for it to achieve its expected effects” (Fairburn and Cooper 2011). It has been described as a component of intervention integrity (Perepletchikova and Kazdin 2005). There are several reasons why therapist competence is important to address, including the responsibility of clinicians to provide their patients with the best possible care, the need to disseminate high-quality evidence-based psychological treatments, and the possible influence of therapist competence on the validity of clinical trials (Fairburn and Cooper 2011; Sharpless and Barber 2009). In general, treatment integrity appears to be an important but often neglected variable in several areas of research and practice, including behavioural interventions (Fryling et al. 2012), primary prevention in schools (Bruhn et al. 2015), and health behaviour (Bellg et al. 2004). The UK Medical Research Council recommends that process evaluations, including assessment of integrity, should be nested within clinical trials of complex interventions to better understand the outcomes and interpret the results of these trials in light of the observed integrity (Craig et al. 2008). In addition, the information that is generated by such integrity checks can be used to differentiate between sites (e.g. in a multi-centre trial) and to further develop and refine treatment (Waltz et al. 1993).

A meta-analytic review showed that in studies targeting depression (*n* = 5), therapist competence was significantly related to outcome with a small-to-medium effect size (*r* = .28) (Webb et al. 2010). These studies included cognitive behavioural therapy (CBT; *n* = 3), interpersonal therapy (*n* = 1), and dynamic psychotherapy (*n* = 1). For example, Kuyken and Tsivrikos (2009) studied the therapy outcomes of 69 patients with depressive disorders who were treated with CBT by one of 18 therapists, whose audio recordings of therapy sessions were evaluated by an expert. The results of this study indicated that greater therapist competence was associated with improved outcomes. Similar findings were reported by Strunk et al. (2010). However, a recent study did not find an association between competence and outcome in a group of 43 CBT therapists and 1247 patients treated for depression and/or anxiety in routine clinical practice (Branson et al. 2015). Although studies may be difficult to compare due to, for example, differences in the instruments used to assess competence, they generally include aspects such as general therapeutic skills, interpersonal skills, effective communication, and flexibly pacing of sessions. In summary, there is evidence for a relationship between competence and outcome in psychotherapy for depression, but this effect is not as consistent or robust as might be expected. This also seems to be the case for more general indicators of therapists’ experience, such as years of clinical experience (Bearman et al. 2013; Mason et al. 2016).

Within the context of MBCT, the effect of teacher competence on treatment outcome has not yet been investigated. The role of the teacher in MBCT is different from the therapist’s role in (individual) psychotherapy, with more emphasis on the patients’ self-efficacy and less knowledge of their personal stories (hence, the use of the word ‘teacher’ rather than ‘therapist’ in this context). However, it is generally assumed that the quality of the teaching is important to ensure that patients receive the intervention as it is intended. Therefore, the aim of the current study was to investigate the possible influence of teacher competence in the delivery of MBCT for recurrent depression on several dimensions relevant to MBCT process and outcome: adherence to treatment (i.e. number of sessions), possible mechanisms of change (rumination, cognitive reactivity, mindfulness, and self-compassion), and key outcome variables (depressive symptoms at post treatment and depressive relapse/recurrence during the 15-month follow-up). The study was part of two multi-centre randomized controlled trials (RCTs) including MBCT as a relapse prevention strategy for patients with recurrent depression (Huijbers et al. 2016, 2015). Both RCTs involved relatively large numbers of MBCT teachers, which allowed us to investigate the possible effect of teacher competence on patient outcomes. We hypothesized that there would be differences between teachers with regard to levels of competence and that higher levels of teacher competence would be associated with better adherence, decreases in rumination and cognitive reactivity, increases in mindfulness and self-compassion, lower levels of depression post treatment, and a lower risk of relapse/recurrence in the year after MBCT.

## Method

### Participants

#### Patients

The patient sample consisted of patients with three or more previous depressive episodes, who were currently in full or partial remission and were using maintenance antidepressants for at least 6 months. The sample of the current study was restricted to patients who were allocated to MBCT. Patients had to have attended at least one session by a teacher whose competence data were available (see below). In total, 241 of the 317 patients in the two trials met these criteria. Seventy-nine (33%) were male. The mean age of the participants was 51.0 (ranging from 23 to 89). The median number of past episodes of depression was 4. All patients provided informed consent to participation in the RCT; the study was approved by the Medical Ethics Committee Arnhem-Nijmegen (nr. 2008/242) for all participating sites. Patients and teachers provided additional informed consent to recording of the sessions on videotape.

#### Teachers

A total number of 21 teachers participated in the trial delivering 113 MBCT classes. Videotapes were available for 15 primary teachers (if classes were taught by two teachers together, we considered the level of the most proficient teacher to reflect the overall competence of the teaching). Seven of the 15 teachers met the advanced criteria of the association of mindfulness-based teachers in the Netherlands and Flanders (www.vmbn.nl), which include a minimum of 150 h of education in MBSR/MBCT (entailing theoretical background, skills practice, supervision, and reflection), a minimum of 3 years of personal meditation practice and attending retreats (minimum of one 10-day retreat or two 5-day retreats), and providing a minimum of two courses per 2 years, and which are in accordance with the UK good practice guidelines (UK Network of Mindfulness-Based Teacher Trainers 2010). All teachers received additional training in the MBCT study protocol during a 3-day training retreat at the start of the project by some of the senior teachers who were involved in previous trials of MBCT (Kuyken et al. 2008; Van Aalderen et al. 2012b). Ongoing peer supervision took place on each site. In addition, the research team organized full-day plenary supervision meetings every 6 months during the intervention phase of the trial, consisting of mindfulness practices, workshops, small group teachings, and plenary discussions about difficulties or practical issues. Table 1 shows the professional and meditation experience of the teachers and assessors.

**Table 1:** Professional and meditation experience of the teachers and assessors

Variable	Teachers (*n* = 15)	Assessors (*n* = 16)
Gender (male/female)	3/12	7/9
Age	*M* = 54 ± 7.2; range 39–64	*M* = 51 ± 9.5; range 34–67
Professional background	Psychologist (8)	Psychologist (7)
	Occupational therapist (3)	Occupational therapist (3)
	Psychiatric nurse (3)	Psychiatrist (2)
	Psychiatrist (1)	Counsellor (2)
		General practitioner (1)
		Other (1)
Clinical experience (years)	*M* = 21 ± 6.5; range 11.5–31	*M* = 19 ± 10.2; range 4.5–35
Years of personal meditation practice	*M* = 9.0 ± 8.0; range 3–35	*M* = 16 ± 9.0; range 6–37
Meditation practice (h/week)	*M* = 4.3 ± 3.3; range 0.5–14	*M* = 3.9 ± 1.2; range 2–7
Number of days spent in retreat	*M* = 57 ± 95; range 0–282	*M* = 161 ± 227; range 11–966
Total amount of personal practice (days)^a^	*M* = 296 ± 231; range 24–845	*M* = 580 ± 532; range 150–2333
Number of MBCT courses taught	*M* = 23 ± 16; range 6–60	*M* = 33 ± 20; range 7–80

^a^The variable is an estimate of the amount of personal practice (lifetime) calculated from the time periods, frequency, and duration of personal home practice (transformed to the corresponding number of 8-h days) added to the number of days spent in silent retreats

### Procedure

This study was based on two parallel randomized controlled trials: the first one comparing the combination of MBCT and antidepressant medication with medication alone (Huijbers et al. 2015) and the second one comparing the combination of MBCT and antidepressant medication with MBCT alone, i.e. with discontinuation of medication (Huijbers et al. 2016). MBCT was largely based on the protocol by Segal et al. (2002b) with some minor adaptations: it consisted of eight weekly sessions of 2.5 (rather than 2) h and included 1 day of silent practice between the sixth and seventh sessions, which originates from the MBSR curriculum (Jon Kabat-Zinn 2013) and is suggested in the most recent version of the MBCT protocol (Segal et al. 2012). Classes were provided at 12 different locations in the Netherlands. All sites used the same materials (protocol, handouts, CDs).

Fifteen MBCT teachers who participated in the RCT as trial teachers provided video recordings of their teaching (two full MBCT courses). The tapes were recorded during the intervention phase of the study between September 2009 and January 2012. From each teacher, two tapes were randomly selected via online list randomization (www.random.org). These were evaluated on competence by two independent raters from a group of 16 assessors.

The assessors were all expert teachers of mindfulness-based interventions. Three assessors also participated as a trial teacher, the others were not involved in the RCTs. All assessors fulfilled the advanced criteria of the association of mindfulness-based teachers in the Netherlands and Flanders. Initially, assessors were invited to take part in a 2-day meeting, including a workshop on the use of the MBI:TAC, led by two of the developers (RC and WK). The workshop consisted of a 2.5-h didactic session in which the background and domains of the MBI:TAC were explained, and assessors’ evaluations were benchmarked using two 30-min (external) video clips of MBCT teaching. Subsequently, the assessors proceeded with the evaluation of the study tapes, working in pairs but starting independently to allow assessment of interrater reliability. Therefore, no discussion between the assessors was allowed during initial assessment. After noting down individual scores, assessors discussed possible differences in scoring and completed a final form with consensus scores for each of the six domains (see below). After each of these sessions, there was a plenary session with the developers of the scale to ask questions and discuss difficulties. About two tapes per teacher were evaluated. Most tapes were evaluated by two assessors, and eight tapes were evaluated by one assessor only. After this initial 2-day meeting, the number of evaluated study tapes was 31.

To maximize the reliability of the MBCT teacher competence ratings, two assessors who were also involved in the initial meeting were invited for further training in using the MBI:TAC and to evaluate 16 additional tapes. As part of this training, their ratings of two non-trial tapes were benchmarked against ratings of one of the developers of the MBI:TAC and discrepancies were discussed (RC). The additional trial tapes were selected from teachers with a minimum of ten participants in their groups (*n* = 8). This resulted in a subsample of eight teachers for whom we had MBI:TAC scores based on four sessions rather than two. Combining the assessments from the initial group meeting (*k* = 31) and the additional tapes from the later assessments (*k* = 16), the final number of evaluated tapes was 47. In addition, we collected notes from the assessors about the process of evaluation to aid our interpretation of the results.

### Measures

#### Teacher Competence

The MBI:TAC was used to assess the competence of the teaching (Crane et al. 2016, 2013, 2012). These criteria have been developed through a consensus process by a group of expert mindfulness trainers in the context of MBSR and MBCT teacher training programmes in the UK, in the period from 2008 to 2012. The MBI:TAC consists of six domains: (1) coverage, pacing, and organization of session curriculum; (2) relational skills; (3) embodiment of mindfulness; (4) guiding mindfulness practices; (5) conveying course themes through interactive inquiry and didactic teaching; and (6) holding of group learning environment. Domains can be scored at six competence levels: incompetent (1), beginner (2), advanced beginner (3), competent (4), proficient (5), and advanced (6), analogous to the Dreyfus and Dreyfus competence scale (Dreyfus and Dreyfus 1986). Within each domain, three to five ‘key features’ are described. The manual, which is freely available online, provides detailed descriptions of these key features and their components and provides examples of how these features might ‘look like’, for each competence level of each domain (Crane et al. 2016). For instance, the example for ‘competent’ in the domain ‘relational skills’ reads “All key features are present to a good level of skill with some minor inconsistencies. Examples include: effective working relationships are generally formed with participants; teacher’s relational style mostly facilitates participants to feel at ease, accepted and appreciated; teacher is confidently attentive to and interested in participants; teacher appropriately brings him/herself into the learning process (mutuality)” (p. 54; version 2016).

Competence was operationalized as the average of the six domains of the MBI:TAC (scores ranging from 1 to 6 per domain), based on the mutually agreed (or if unavailable, the individual) scores, yielding a single competence score per teacher.

An early study of the psychometric properties of the MBI:TAC suggests good reliability in terms of good overall agreement (*r* = .81) and substantial agreement for the individual domains (intraclass correlation coefficients (ICCs) ranging from .60 to .81) (Crane et al. 2013). Good face validity, construct validity, and concurrent validity were reported in terms of between-domain correlations (ranging from .60 to .84) and significant differences between teachers in their first year of training and those in their second year or beyond.

#### Possible Mediators

##### Rumination

Rumination was measured with the ‘brooding’ subscale of the extended version of the Ruminative Response Scale (RRS-EXT) (Treynor et al. 2003). The authors reported adequate internal consistency (*α* = .79) and test–retest stability (*α* = .62, 1-year time interval) for the brooding subscale, which consists of five items. We selected the brooding subscale because over time, brooding has been related to higher levels of depression, whereas the reflection subscale has been linked to lower levels of depression (Treynor et al. 2003). The internal consistency in the current study was *α* = .75.

##### Cognitive Reactivity

Cognitive reactivity was assessed using the Leiden Index of Depression Sensitivity-Revised (LEIDS-R) (Van der Does 2002). This scale consists of 34 items comprising six subscales of five or six items, which had the following internal consistencies in the current study: hopelessness/suicidality (*α* = .83), acceptance/coping (*α* = .63), aggression (*α* = .73), control/perfectionism (*α* = .64), risk aversion (*α* = .70), and rumination (*α* = .73). The internal consistency of the total score was *α* = .85.

##### Mindfulness Skills

Mindfulness skills were assessed using the Five Facet Mindfulness Questionnaire (FFMQ), consisting of 39 items divided into the subscales observing, describing, acting with awareness, non-judging, and non-reactivity (Baer et al. 2006). The FFMQ has been found reliable and valid in a Dutch sample of depressed individuals (Bohlmeijer et al. 2011). In the current study, the following internal consistencies were found: observing (*α* = .74), describing (*α* = .89), acting with awareness (*α* = .86), non-judging (*α* = .88), non-reactivity (*α* = .79), and total score (*α* = .87).

##### Self-Compassion

Self-compassion was measured with the Self-Compassion Scale (SCS) (Neff 2003). The SCS has 26 items measuring three concepts that are related to self-compassion: (a) self-kindness versus self-judgement, (b) common humanity versus isolation, and (c) mindfulness versus over-identification. Good validity for the SCS has been reported (Neff 2003). In the current study, the following internal consistencies were found: self-kindness (*α* = .73), self-judgement (*α* = .79), common humanity (*α* = .70), isolation (*α* = .76), mindfulness (*α* = .73), over-identification (*α* = .68), and total score (*α* = .72).

#### Outcome Measures

##### Depressive Symptoms

The Inventory of Depressive Symptomatology-Clinician Rated (IDS-C) was used to assess the severity of depressive symptoms at post treatment (Rush et al. 1996). This clinician-rated scale consists of 30 items assessing the criterion symptoms designated by the Diagnostic and Statistical Manual of Mental Disorders-4th edition (DSM-IV) (American Psychiatric Association 2000) for major depressive disorder for the prior 7 days. The IDS-C has good psychometric qualities (Rush et al. 1996; Trivedi et al. 2004). The IDS-C was administered by independent, trained research assistants (Huijbers et al. 2012). Cronbach’s alpha in the current study was .84 for the baseline assessment and .89 at post treatment.

##### Depressive Relapse/Recurrence

Relapse/recurrence of depression was defined as meeting the DSM-IV criteria for a depressive episode during the 15-month study period using the Structured Clinical Interview for DSM disorders I (SCID-I) (First et al. 1996). See Huijbers et al. (2012) for more details. Fair-to-good reliability has been reported for SCID-I in depressed samples (Lobbestael et al. 2011; Zanarini et al. 2000). In the current study, the interrater reliability between the first and second ratings was found to be substantial (kappa = 0.70, *p* = .001, 95% CI 0.456–0.942).

### Data Analyses

To assess the interrater reliability of the MBI:TAC, ICCs were calculated using a two-way random consistency model with single measures, based on the independent ratings of two assessors per videotape (*n* = 42). For interpreting the strength of these ICCs, the following cut-off points are used: <0.00 (poor), 0.00–0.20 (slight), 0.21–0.40 (fair), 0.41–0.60 (moderate), 0.61–0.80 (substantial), and 0.81–1.00 (almost perfect) (Landis and Koch 1977). In addition, exact agreement and agreement including adjacent scores were calculated per domain. As an external validity check, we calculated the correlations between the mean MBI:TAC scores and teacher experience (years of clinical experience, personal mindfulness practice, and number of MBCT courses) and between MBI:TAC scores and teachers’ self-reported mindfulness skills.

For all analyses, we performed complete case analyses (see Table 2 for the numbers). Probability values lower than .05 (two-tailed) were considered significant in all analyses. We used separate analyses for each outcome measure. Linear regression analyses were used to examine the relationship between teacher competence and patients’ MBCT adherence, with the MBI:TAC score as a predictor of the number of sessions attended by participants. To investigate the possible association between teacher competence and continuous process and outcome measures, firstly, multilevel analyses were used to investigate the amount of variance in the outcome measures at the level of the participants (*n* = 241) that could be explained at the level of the teacher (*n* = 15). This was expressed as an ICC, calculated as teacher variance / (teacher variance + residual variance). Baseline scores of the process and outcome variables were included as covariates in the respective analyses. In addition, we included age, gender, number of past episodes (log-transformed), depressive symptomatology at baseline (for the outcomes other than depressive symptomatology), and previous CBT experience (yes/no) as covariates. Subsequently, MBI:TAC scores were added to the model to test whether teacher competence would have an additional value in predicting the variance in outcomes. Cox regression analysis was performed to examine the association between teacher competence and depressive relapse/recurrence during the 15-month study period. In case of dropout of the study, participants were censored before dropout, and others were censored at the end of the study period. Analyses were performed both with and without covariates.

**Table 2:** Means and standard deviations of the continuous mediator and outcome variables

Variable	Pre-treatment mean (SD), *n*	Post-treatment mean (SD), *n*	*F*(*df*)	Effect size (*d*)
RRS-Br	11.0 (3.0), 234	9.8 (3.2), 186	28.22 (1180) ***	0.39
LEIDS-R	76.7 (14.3), 233	74.0 (15.0), 186	8.24 (1180) **	0.18
FFMQ	117.1 (15.5), 232	127.9 (16.9), 185	84.32 (1177) ***	0.67
SCS	86.9 (14.4), 232	93.1 (15.2), 184	37.26 (1176) ***	0.42
IDS-C	12.8 (9.7), 241	13.2 (10.9), 201	1.48 (1200)	−0.09

*RRS-Br* Ruminative Response Scale-Brooding Subscale, *LEIDS-R* Leiden Index of Depression Sensitivity-Revised, *FFMQ* Five Facet Mindfulness Questionnaire, *SCS* Self-Compassion Scale, *IDS-C* Inventory of Depressive Symptomatology-Clinician Rated

**p < .01; ***p < .001

Sensitivity analyses were performed for the subsample of teachers for whom we had four videotapes evaluated (*n* = 8), i.e. for whom we expected the MBI:TAC estimates to be more reliable than for teachers who were evaluated on two videotapes. We also performed all analyses on the original sample (*n* = 15, assessments from the first two videotapes only). However, as the pattern of results in both sensitivity analyses was very similar to the complete sample (*n* = 15, using all available assessments), we only report the results from the complete sample. Another set of sensitivity analyses was performed using the average rather than the mutually agreed MBI:TAC score as a predictor. However, this led to highly similar results, and the correlation between the agreed and average scores was very high (*r* = .99) so we only reported the results based on mutually agreed scores.

Exploratory analyses were performed to differentiate between the individual domains of the MBI:TAC and to examine indicators of teachers’ experience (years of practice as a clinician, the number of MBCT courses taught, and the total amount of personal practice) as predictors of all process and outcome variables. Personal practice was calculated from teachers’ registrations (retrospectively) of the time periods, frequency, and duration of personal home practice, transformed to the corresponding number of 8-h days, added to the number of days spent in silent retreats. For these analyses, we used the same model as for the primary analyses (i.e. multilevel model for the continuous variables and Cox regression analysis for relapse/recurrence).

## Results

### Treatment Outcome

Table 2 shows the means and standard deviations of the continuous mediator and outcome variables. Cognitive reactivity and brooding decreased significantly from pre to post treatment with small effect sizes, whereas mindfulness skills and self-compassion increased significantly with medium and small effect sizes, respectively. Depressive symptoms did not change from pre to post treatment. Of the 241 participants, 115 (47.7%) experienced a relapse/recurrence in the observed time period. For the completer sample (attending at least four sessions; *n* = 221), the results were similar.

### Levels of Teacher Competence

The mean MBI:TAC score (averaged across tapes; *n* = 15) was 3.53 (SD 0.92, range 2.00–5.15). The means and standard deviations for the individual domains were as follows: 3.58 ± 0.91 (coverage/organization), 3.62 ± 0.94 (relational skills), 3.61 ± 1.04 (embodiment), 3.54 ± 0.95 (guiding practices), 3.53 ± 1.13 (inquiry and teaching), and 3.29 ± 1.00 (group management). This suggests that the competence scores of the evaluated sessions were, on average, between the ‘advanced beginner’ and ‘competent’ level. In terms of discrete competence levels, none of the teachers was incompetent, two teachers (13%) were characterized as beginners, six (40%) as advanced beginners, four (27%) as competent, three (20%) as proficient, and none as advanced.

### Reliability and Validity of the MBI:TAC

The internal consistency of the MBI:TAC was high (Cronbach’s alpha = .96). The ICCs of the six domains were as follows: 0.55 (moderate) for domain 1 ‘coverage/pacing’, 0.67 (substantial) for domain 2 ‘relational skills’, 0.45 (moderate) for domain 3 ‘embodiment’, 0.68 (substantial) for domain 4 ‘guiding practices’, 0.63 (substantial) for domain 5 ‘inquiry and teaching’, and 0.58 (moderate) for domain 6 ‘group management’. Low agreement was observed for the percentages of exact agreement, ranging between 29 and 40%. When adjacent scores were included as agreement, percentages ranged between 69 and 88%.

Correlations between the domains of the MBI:TAC were high, ranging from .76 to .94 (all *p* values <.01). Table 3 shows the correlations between the MBI:TAC mean score and indicators of teachers’ experience and mindfulness skills. MBI:TAC scores were not significantly correlated with clinical experience (in years). A trend was observed between the personal meditation practice and the MBI:TAC domain ‘group management’. The number of MBCT courses was not significantly correlated with MBI:TAC scores, but correlations were all in the positive direction. Unexpectedly, albeit not significant, correlations between the MBI:TAC scores and FFMQ total scores were all in the negative direction, ranging between −.01 and −.56.

**Table 3:** Correlations (Pearson’s *r*) between the MBI:TAC domains and indicators of teachers’ experience and mindfulness skills

Variable	Clinical experience (years)	Total amount of personal practice (days)	Number of MBCT courses	Mindfulness skills (FFMQ total)
MBI:TAC (mean)	.03	.39	.32	−.43
(1) Coverage/organization	.22	.36	.45	−.01
(2) Relational skills	−.02	.39	.34	−.38
(3) Embodiment	.17	.35	.30	−.48
(4) Guiding practices	−.13	.40	.25	−.56^a^
(5) Inquiry and teaching	−.10	.31	.15	−.52
(6) Group management	.08	.45^a^	.32	−.19
	*N* = 14	*N* = 15	*N* = 14	*N* = 10

^a^The trend towards significance (*p* values between .05 and .10)

### Teacher Competence and Adherence

The attrition rate, defined as attending fewer than four out of eight sessions in accordance with previous trials (Kuyken et al. 2008; Teasdale et al. 2000), was 8% (*N* = 20/241). The median number of sessions was 7. Linear regression analysis with the covariates in the first block and MBI:TAC score as a predictor in the second block showed that MBI:TAC did not improve the model for the number of sessions attended as an outcome measure (model 1: *R* ^2^ = .023, *F*(5,235) = 1.11, *p* = .358; model 2: *R* ^2^ change = .000, *F* change (1,234) = 0.92, *p* = .483). Similar results were obtained for analysis without covariates.

### Teacher Competence and Possible Mediators

**Fig. 1:**
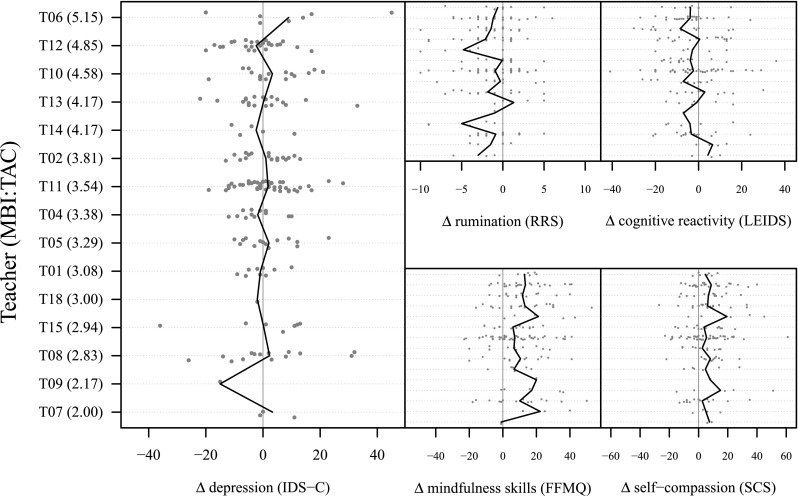
Changes in depressive symptoms, rumination, cognitive reactivity, mindfulness skills, and self-compassion from pre to post treatment, grouped by the mean teacher competence score from lowest to highest. The *solid line* represents the mean

Figure 1 shows the changes in rumination, cognitive reactivity, mindfulness skills, and self-compassion from pre to post treatment, grouped by the mean teacher competence score from lowest to highest. Based on visual inspection, the largely vertical orientation for the solid lines (indicating the group mean change scores) suggests that participants from different teachers did not have different outcomes. The results of the multilevel analyses indicated that the variance explained by the teacher was negligible for all outcomes (ICC values <.01). Analyses without the covariates, except for the baseline score of the outcome measure, yielded similar results (all *p* values >.1).

Adding the MBI:TAC score to the model did not explain variance in any of the outcome measures (all *p* values ≥.1). Analyses without the covariates, except for the baseline score of the outcome measure, yielded similar results.

Exploratory analyses with the individual MBI:TAC domain scores showed that there were no significant associations between these domain scores and changes in the possible mediators. Exploratory analyses with years of clinical practice, the number of MBCT courses taught, and the total amount of personal practice as individual predictors did not show a relationship with any of the mediators either.

### Teacher Competence and Outcomes: Depressive Symptoms and Relapse/Recurrence

Figure 1 shows the changes in depression severity from pre to post treatment grouped by the mean teacher competence score from lowest to highest. Again, visual inspection of the solid lines showed a vertical orientation, indicating that the mean difference in depression severity between pre and post treatment did not differ between patients of different teachers. The pattern of results was similar to that of the possible mediators: the variance in changes in depression severity could neither be explained by the teacher nor by the MBI:TAC score (including the individual domains). Depression outcomes could not be explained by years of clinical practice, number of MBCT courses taught, and personal practice of the teacher as individual predictors either.

Cox regression analysis with MBI:TAC as a predictor for relapse with age, gender, number of past episodes (log transformed), baseline depression score, and CBT experience as covariates showed that the MBI:TAC score did not make a significant contribution to the model (hazard ratio = 1.07, 95% CI 0.83 to 1.38, *p* = .60). The model without covariates yielded similar results (hazard ratio = 1.00, 95% CI 0.78 to 1.28, *p* = .99). Exploratory analyses with (a) the individual MBI:TAC domain scores and (b) years of clinical practice, number of MBCT courses taught, and personal practice as individual predictors did not show any relationship with relapse/recurrence either.

## Discussion

In the current study, we found that teacher competence as assessed by the MBI:TAC was not associated with patients’ adherence to MBCT sessions, changes in possible mediating variables (rumination, cognitive reactivity, mindfulness, and self-compassion), or depression severity from pre to post treatment, or with relapse/recurrence during the 15-month follow-up. Other indices of teacher competence, such as their level of experience as measured by the number of MBCT classes they had led, did not predict mediator and outcome variables either.

This study systematically examined the relation between competence and outcome in MBCT. Interestingly, earlier studies of CBT in depression did indicate therapist competence to be related to treatment outcome (e.g. Kuyken and Tsivrikos 2009). However, a more recent study of 43 CBT therapists and 1247 patients in routine clinical practice did not show an association between therapist competence and treatment outcome either (Branson et al. 2015). Our own study also took place in routine clinical care, with 12 centres around the Netherlands participating. The 15 teachers included in our study were not taught or supervised by the developers of MBCT as in earlier trials (Kuyken et al. 2016) and showed a wider range of competence levels, including more beginner and advanced beginner scores than in other trials reporting teacher competence (Kuyken et al. 2015, 2008; Williams et al. 2014). That being said, the lowest and highest levels of competence were still underrepresented in the current study. There were only two teachers characterized as ‘beginner’, and none of the teachers were, on average, characterized as advanced. This may have caused some restriction of range, possibly undermining any association between competence and outcome.

Another possible explanation for the absence of a relationship between competence and outcome in MBCT is that the MBCT program might ‘carry itself’. Participants are given standardized pre-recorded mindfulness practices, invited to take full responsibility for themselves and to develop self-efficacy. This is also reflected in the strong emphasis on doing homework practices and applying mindfulness to daily life. Most of the work takes place between the sessions (about 6 h of home practice per week) rather than within the sessions (2.5 h). Previous studies have suggested that the patients’ amount of home practice is related to the risk of relapse/recurrence (Crane et al. 2014) and a decrease of depressive symptoms (Van Aalderen et al. 2012b) after MBCT and to a decrease in rumination (Ramel et al. 2004) and an increase in wellbeing (Carmody and Baer 2008) after MBSR. Thus, participants’ willingness to engage in practice and explore their experiences both in and between the sessions may be more important to change than MBCT teacher competence. In this and most other studies, rates of patient engagement and adherence tend to be high. Therefore, the relationship between teacher and participant is possibly less influential than in other types of psychotherapy. Furthermore, MBCT is delivered in groups with group members providing a sense of group support which may decrease the importance of the therapeutic relationship. This has been suggested by several qualitative studies of MBCT participants (e.g. Allen et al. 2009; Mason and Hargreaves 2001), including one study that focused exclusively on the role of the teacher in MBCT (Van Aalderen et al. 2012a). Interestingly, all participants in this latter study mentioned the importance of peer support whereas this was mentioned by only a few teachers.

An additional explanation for the absence of a relationship might be the relatively uniform delivery of the intervention within the trial. MBCT is highly standardized (especially in the context of an RCT). All study participants received the same materials, and participants’ home practice was led through the same mindfulness practice CDs.

Another striking finding of our study is the complexity and difficulty of assessing the practitioner competence in a complex intervention. The reliability of the MBI:TAC in our study was lower than that reported by the developers of the instrument (Crane et al. 2013). ICCs were moderate to substantial, suggesting that a considerable amount of variance can be attributed to differences in ratings between assessors. There are several explanations for the relatively low agreement between assessors in our study. Unlike the assessors in the study by Crane et al. (2013), who collaborated in the development of the MBI:TAC for several years, the assessors in our study had no previous experience with the instrument, were trained in MBCT at different institutes, were less acquainted with each other, and evaluated teachers of whose teaching they had no or little prior knowledge. A study by Keen and Freeston (2008) indicated that reliable assessment of competence is complex and resource intensive: in order to obtain adequate reliability for CBT competencies, 19 videotapes of one therapist evaluated by two assessors would be necessary. Although we increased the number of evaluated tapes from 2 to 4 for a substantial number of the teachers to begin to address this issue, we were not able to meet this stringent target. Therefore, the results of our study should be considered with caution. On the other hand, several indicators of teachers’ experience (years of practice as a clinician, number of MBCT courses taught, and personal practice) were not associated with treatment outcome either, which supports the robustness of our findings independent of the MBI:TAC ratings.

An unexpected observation was that the teachers’ self-reported levels of mindfulness skills, as measured with the FFMQ, were negatively (albeit not significantly) correlated with the MBI:TAC domains. This may suggest that teachers who are considered to be more competent are more aware of their lack of mindfulness than those considered less competent. However, this finding should be interpreted with caution as data on mindfulness skills were only available for 10/15 teachers.

Interestingly, a study showed that patients’ perceptions of the therapeutic alliance are related to outcome in individual psychotherapies, but that these perceptions did not necessarily match those of the therapists (Horvath et al. 2011). In future studies, it would be interesting to use triangulation, for example by using self-reports and experts’ and patients’ perspectives on teacher competence in mindfulness-based interventions, to see how these correlate and possibly (differentially) predict outcome.

On the basis of the notes from the expert teacher competence assessors, we identified some challenges in the process of assessing competence. First, the constituent trials often used two teachers and the presence of a co-teacher can significantly impact the teaching of the other. Although in the current study only a minority of patients attended MBCT provided by two teachers (28%) and the competence ratings of these teachers differed at less than 1 point (on a scale from 1 to 6), we cannot rule out that the presence of co-teachers may have influenced teacher competence and, consequently, the results of this study. Thus, the possible impact of a co-teacher should be taken into account when assessing the competence in future studies. A second challenge that came up was the difficulty of distinguishing amongst the different domains, i.e. coverage/organization, relational skills, embodiment, guiding practices, inquiry and teaching, and group management. For example, the assessors noted in a particular case that the lack of organization of the session seemed to stem from a lack of embodiment. The overlapping of domains sometimes led to confusion with regard to choosing whether to include the information in one domain or the other, or in both. Furthermore, the numerous and detailed descriptions of the domains (including key features and corresponding criteria) seemed challenging. Some elements or criteria may speak strongly to one assessor, whereas other elements may be more important to another assessor, resulting in different ‘weighting’ of the criteria. The assessors noticed that despite having similar overall impressions of a teacher, their individual scores could be different. In addition, the MBI:TAC criteria emphasize the use of interactive dialogue as a key method in mindfulness-based teaching, for example to explore participants’ experiences or to convey course themes. However, in some cases, the assessors observed types of teaching, such as active listening or presenting psycho-education (without dialogue) in a very inspiring way, which seemed to deepen the learning process as well. In these cases, using the criteria led to lower scoring than when a more general impression of the teaching was followed.

### Limitations

Some limitations of the current study should be considered when interpreting the results. The data were drawn from a relatively homogeneous sample of recurrently depressed patients in remission who had been using antidepressant medication for a relatively long time. Therefore, the results may not be generalizable to other populations. In addition, the fact that participants were in remission at baseline precluded large pre- to post-treatment changes in depressive symptoms that can be observed in acute treatment studies. Teacher effects may be more pronounced when MBCT is provided as a treatment for acute symptoms of depression or anxiety, for example. Other limitations include the lack of randomization with regard to teacher competence (i.e. post hoc comparisons); the lack of cultural diversity in all three samples (patients, teachers, and assessors), which mainly comprised white Caucasian persons; and the use of questionnaires and interviews that may be biased due to their inherent subjectivity.

In conclusion, we did not find robust effects of teacher competence on several outcomes of MBCT. Explanations for the absence of such an association might be the standardized delivery of MBCT, the importance of participant-related factors, the difficulties in assessing teacher competence, and a relatively small selection of videotapes. It is possible that the role of the teacher relative to the curriculum, the group, the mindfulness home practice, and the participants themselves is overestimated. However, as this is an area that is under investigation, we encourage other researchers who conduct trials of mindfulness-based interventions to systematically assess teacher competence and its possible influence on treatment outcome so that the field can develop an understanding of this nuanced and complex area.
